# A Mathematical Model to Study the Effectiveness of Some of the Strategies Adopted in Curtailing the Spread of COVID-19

**DOI:** 10.1155/2020/5248569

**Published:** 2020-10-13

**Authors:** Isa Abdullahi Baba, Bashir Abdullahi Baba, Parvaneh Esmaili

**Affiliations:** ^1^Bayero University, Kano, Nigeria; ^2^Near East University, TRNC, Turkey

## Abstract

In this paper, we developed a model that suggests the use of robots in identifying COVID-19-positive patients and which studied the effectiveness of the government policy of prohibiting migration of individuals into their countries especially from those countries that were known to have COVID-19 epidemic. Two compartmental models consisting of two equations each were constructed. The models studied the use of robots for the identification of COVID-19-positive patients. The effect of migration ban strategy was also studied. Four biologically meaningful equilibrium points were found. Their local stability analysis was also carried out. Numerical simulations were carried out, and the most effective strategy to curtail the spread of the disease was shown.

## 1. Introduction

A mathematical model can be defined as a method of describing a system using mathematical language and notions in order to facilitate a proper explanation of the system or to know the effects of various components on the system and to be able to predict patterns of behavior [1]. The procedure of constructing a mathematical model is called mathematical modeling [2]. Mathematical modeling plays a vital role in the investigation of problems that affect our day-to-day life more specifically in the field of medicine and biological sciences. For example, mathematical models gave insight into tumorigenesis [3], avascular tumor growth [3], tumor metabolism during invasion [4], cell migration [5], angiogenesis [3, 6, 7], tumor evolution [3, 8], and cancer stem cells [9].

Robots are man-made computerized machines which perform tasks on human commands or by themselves and seek to make work easier for human beings. The research of robot modeling provides better understanding of the interaction of robots and its environment [10]. These models can help us learn the main factors of the behaviors of the system and also help us in improving the understanding of the properties of robot collective behaviors that emerged. The methodology known as probabilistic robotics for programing robots was described by Thrun [11]. The probabilistic method for the multiple robot coordination was presented by Burgard et al. [12]; the method considers both the utility of target points and the cost of reaching a target point. The algorithm for localization of collaborative mobile robots was presented by Fox et al. [13]; the method was successfully able to use the sample-based version of Markov localization to localize mobile robots in any time fashion.

Towards the end of December 2019, a massive case of pneumonia from an unknown source was found in China which spread throughout the country within a month. Later, the pathogen of the disease was identified by means of molecular methods as a novel coronavirus and it was named as novel coronavirus 2019 (2019 n-CoV); however, in February 2020, the World Health Organization (WHO) renamed the epidemic disease as coronavirus disease 2019 (COVID-19) [14–17]. Patients with this illness are frequently presented with cough, fever, and shortness of breath within 2 to 14 days after exposure [18]. As of March 23, 2020, there had been 332,930 confirmed cases of COVID-19 reported globally, and 14,510 deaths had been reported [19]. In recognition of the widespread global transmission of COVID-19, the World Health Organization declared COVID-19 to be a pandemic on March 11, 2020 [20].

There is no treatment and vaccine against the infection yet, but the standard recommendation to prevent infection spread at the individual level is the compliance with orientation strategies that include reporting of suspected case, self-isolation, regular hand washing using sanitizers, covering mouth and nose when coughing and sneezing, and avoiding contact with anyone showing symptoms of respiratory illness such as coughing and sneezing [21].

However, to curtail the spread of the infection, many governments undertake the following policies: prohibit migration of individuals into their countries especially from those countries that were known to have COVID-19 epidemic, wide public orientation on distancing from public gatherings that include social and religious, banning both the local and international air trip, closing both public and private institutions that may attract large gathering, contact tracing and isolation of infected individuals, providing sanitizers at public domains like markets and car park, fumigating the area where an infected individual comes from, and to a large extent imposing stay at home curfew.

Considering the current disastrous situation, robots are well suited for caring for the well-being of COVID-19 patients thus replacing or at least sharing the workload of the medical staff in hospitals under oversaturated conditions. A number of robotic systems are used for medical support in hospitals today [22]. In China, robots have been assigned multiple tasks to minimize the spread of COVID-19, such as utilizing them for cleaning and food preparation jobs in infected areas hazardous for humans. This study is one of the first studies which highlight the importance of robotics in hospitals and healthcare facilities specially concerned with the COVID-19 outbreak.

Looking at the nature of the spread of COVID-19, i.e., from an infected to a healthy person through the eye, nose, and mouth, via droplets produced upon coughing or sneezing and contact with contaminated surfaces, objects, or items of personal use [23], this put the life of medical practitioners at risk. The prime utilization of robots is to minimize person-to-person contact and to ensure cleaning, sterilization, and support in hospitals especially in carrying out tests. This will result in minimizing the life threat to medical staff and doctors taking an active role in the management of the COVID-19 pandemic. The intention of the present research is to highlight the importance of medical robotics in general and then to connect its utilization with the perspective of COVID-19 management so that the hospital management can direct themselves to maximize the use of medical robots for various medical procedures. Also, some of the government policies were excellent in curtailing the spread of the disease, but employing a single policy may not be very much effective. In this paper, we developed a model that suggests the use of robots in identifying COVID-19-positive patients and which studies the effectiveness of the government policy of prohibiting migration of individuals into their countries especially from those countries that were known to have COVID-19 epidemic.

The paper is arranged as follows.  Section 1 gives the introduction; Section 2 studies the model formulation and meaning of parameters and variables; Section 3 gives the stability analysis of the equilibria; Section 4 gives the detailed numerical simulations and studies the effect of the control measures taken; and finally, Section 5 gives the summary and conclusion of the paper.

## 2. Model Formulation

We consider a compartmental model consisting of two equations; the first equation illustrates the dynamics of infected individual population. The population of infected individuals increases by introducing new infected individuals by one of the methods of infection *αI* or by migration of infected individuals from another country *C*(*I*). It is reduced by death due to infection *μI* or when a robot identifies an infected individual *βRI*, who will subsequently be taken into isolation (when he shows no sign of infection) or quarantine (when he shows a sign of infection). The second compartment illustrates the dynamics of the robot population. It increases by constant introduction of newly formed robots *γ* and reduces when a robot stops functioning *θR*.  Table 1 gives the meaning of the variables and parameters.

Then,

(1) $$\begin{array}{c} \left\{\begin{array}{c} \frac{dI}{dt}=\alpha I-\beta RI+C\left(I\right)-\mu I,\\ \frac{dR}{dt}=\gamma -\theta R, \end{array}\right. \end{array}$$

where

(2) $$\begin{array}{c} C\left(I\right)=\left\{\begin{array}{c} C,C>0,\\ \frac{C}{I},C\geq 0. \end{array}\right. \end{array}$$

*C*(*I*) = *C* when there are many infected human migrants, and *C*(*I*) = *C*/*I* when there are few infected human migrants.

## 3. Analysis of the Model

We consider and analyze two models in this paper:

(3) $$\begin{array}{c} \left\{\begin{array}{c} \frac{dI}{dt}=\alpha I-\beta RI+C-\mu I,\\ \frac{dR}{dt}=\gamma -\theta R. \end{array}\right. \end{array}$$

In model (3), there is no government restriction on the migration of individuals into the country, as such the infective population increases by *C* (many infected human migrants).

(4) $$\begin{array}{c} \left\{\begin{array}{c} \frac{dI}{dt}=\alpha I-\beta RI+\frac{C}{I}-\mu I,\\ \frac{dR}{dt}=\gamma -\theta R. \end{array}\right. \end{array}$$

In model (4), there is government restriction on the migration of individuals into the country, as such the infective population increases by *C*/*I* (few infected human migrants).

### 3.1. Equilibrium Points and Stability Analysis of Model 1

Let

(5) $$\begin{array}{c} f_{1}\left(I,R\right)=\alpha I-\beta RI+C\left(I\right)-\mu I,\\ f_{2}\left(I,R\right)=\frac{dR}{dt}=\gamma -\theta R,\\ \text{equating }f_{1}=f_{2}=0. \end{array}$$

If *R* = 0,

(6) $$\begin{array}{c} \left(\alpha -\mu \right)I^{*}=-C,\\ I^{*}=\frac{C}{\mu -\alpha }. \end{array}$$

Therefore, this equilibrium point *E*_0_ = (*I*^∗^, 0) = (*C*/*μ* − *α*, 0) exists (biologically meaningful) if *μ* > *α*.

This equilibrium entails a situation whereby there are infected individuals in the population but there are no available robots to identify them. In this case, asymptomatic cases cannot be identified as such the disease will continue to spread.

If *I* ≠ 0 and *R* ≠ 0, then

(7) $$\begin{array}{c} E_{1}=\left(I^{*},R^{*}\right)=\left[\frac{C}{\left(\beta \gamma /\theta \right)+\mu -\alpha },\frac{\gamma }{\theta }\right]. \end{array}$$

Therefore, this equilibrium point exists (biologically meaningful) if (*βγ*/*θ*) + *μ* > *α*.

This equilibrium entails a situation whereby both infected individuals and robots are present in the population. It is expected that infected individuals will be identified by the robots, and hence, the disease spread can be curtailed.

**Theorem 1 .**

The equilibrium point *E*_0_ is locally asymptotically stable when *α*/*μ* < 1.

**Proof**

Consider the Jacobian matrix of (3)

(8) $$\begin{array}{c} J\left(I,R\right)=\left(\begin{array}{cc} \alpha -\beta R-\mu & -\beta I\\ 0 & -\theta \end{array}\right). \end{array}$$

The characteristic equation of the Jacobian matrix is in general given as

(9) $$\begin{array}{c} \lambda^{2}+t_{r}\left(J\right)\lambda +\det\left(J\right)=0. \end{array}$$

The solution is locally asymptotically stable if *t*_*r*_(*J*)*λ* < 0 and det(*J*) > 0. So both the eigenvalues have negative real parts.

Now, substituting *E*_0_ = (*C*/*α* − *μ*, 0) in (8), we get

(10) $$\begin{array}{c} J\left(\frac{C}{\mu -\alpha },0\right)=\left(\begin{array}{cc} \alpha -\mu & -\beta C/\alpha -\mu \\ 0 & -\theta \end{array}\right),\\ t_{r}\left(J\left(\frac{C}{\mu -\alpha },0\right)\right)=\alpha -\mu -\theta ,\\ t_{r}\left(J\left(\frac{C}{\alpha -\mu },0\right)\right)<0\;\text{if }\alpha <\mu +\theta ,\frac{\alpha }{\mu +\theta }<1,\\ \det\left(\;J\left(\frac{C}{\alpha -\mu },0\right)\right)=-\theta \left(\alpha -\mu \right),\\ \det\left(\;J\left(\frac{C}{\alpha -\mu },0\right)\right)>0\;\text{if }\alpha -\mu <0,\frac{\alpha \;}{\mu }<1. \end{array}$$

**Theorem 2 .**

The equilibrium point *E*_1_ is locally asymptotically stable when (*μθ* + *βγ* + *θ*^2^)/*αθ* > 1.

**Proof**

Substitute *E*_1_ = (*Cθ*/(*βγ* + *μθ* − *αθ*), *γ*/*θ*) in (8), we get

(11) $$\begin{array}{c} J\left(\frac{C\theta }{\beta \gamma +\mu \theta -\alpha \theta },\frac{\gamma }{\theta }\right)=\left(\begin{array}{cc} \alpha -\mu -\beta \frac{\gamma }{\theta } & \frac{-\beta C\theta }{\beta \gamma +\mu \theta -\alpha \theta }\\ 0 & -\theta \end{array}\right),\\ t_{r}\left(J^{*}\right)=t_{r}\left(J\left(\frac{C\theta }{\beta \gamma +\mu \theta -\alpha \theta },\frac{\gamma }{\theta }\right)\right)=\alpha -\mu -\frac{\beta \gamma }{\theta }-\theta ,\\ t_{r}\left(J\left(\frac{C\theta }{\beta \gamma +\mu \theta -\alpha \theta },\frac{\gamma }{\theta }\right)\right)<0\;\text{if }\alpha <\mu +\frac{\beta \gamma }{\theta }+\theta ,\frac{\alpha \theta }{\mu \theta +\beta \gamma +\theta^{2}}<1,\frac{\mu \theta +\beta \gamma +\theta^{2}}{\alpha \theta }>1,\\ \det\left(J^{*}\right)=\det\left(J\left(\frac{C\theta }{\beta \gamma +\mu \theta -\alpha \theta },\frac{\gamma }{\theta }\right)\right)=-\theta \left[\alpha -\mu -\frac{\beta \gamma }{\theta }\right],\\ \det\left(J\left(\frac{C\theta }{\beta \gamma +\mu \theta -\alpha \theta },\frac{\gamma }{\theta }\right)\right)>0\;\text{if }\alpha -\mu -\frac{\beta \gamma }{\theta }<0,\frac{\alpha \theta }{\beta \gamma +\mu \theta }<1,\frac{\mu \theta +\beta \gamma +\theta^{2}}{\alpha \theta }>1. \end{array}$$

### 3.2. Equilibrium Points and Stability Analysis of Model 2

Let

(12) $$\begin{array}{c} f_{3}\left(I,R\right)=\alpha I-\beta RI+\frac{C}{I}-\mu I,\\ f_{4}\left(I,R\right)=\frac{dR}{dt}=\gamma -\theta R,\\ \text{equating }f_{3}=f_{4}=0. \end{array}$$

If *R* = 0, we have

(13) $$\begin{array}{c} \alpha I+\frac{C}{I}-\mu I=0,\\ \alpha I^{2}-\mu I^{2}+C=0,\\ I^{2}=\frac{C}{\mu -\alpha },\\ I=\pm \sqrt{\frac{C}{\mu -\alpha }}. \end{array}$$

But biologically, $I=-\sqrt{C/\mu -\alpha }$ is not meaningful.

So, we consider $I^{*}=\sqrt{C/\mu -\alpha }$, with *μ* > *α*.

(14) $$\begin{array}{c} E_{2}=\left(I^{*},0\right)=\left(\sqrt{\frac{C}{\mu -\alpha },0}\right). \end{array}$$

This equilibrium entails a situation whereby infected individuals and migration restrictions are present in the population. It is expected that the population of infected individuals will not increase much.

Now, when both *I*^∗^ ≠ 0 and *R*^∗^ ≠ 0, we have

(15) $$\begin{array}{c} R^{*}=\frac{\gamma }{\theta },\\ \alpha I-\beta \frac{\gamma }{\theta }I+\frac{C}{I}-\mu I=0,\\ \alpha I^{2}-\beta \frac{\gamma }{\theta }I^{2}+C-\mu I^{2}=0,\\ -C=I^{2}\left[\alpha -\frac{\beta \gamma }{\theta }-\mu \right],\\ I^{*}=\pm \sqrt{\frac{C}{\left(\beta \gamma /\theta \right)+\mu -\alpha }.} \end{array}$$

Again, $I^{*}=-\sqrt{C/\left(\left(\beta \gamma /\theta \right)+\mu -\alpha \right)}$ is not biologically meaningful; hence, we consider $I^{*}=\sqrt{C/\left(\left(\beta \gamma /\theta \right)+\mu -\alpha \right)}$ which exists only when

(16) $$\begin{array}{c} \frac{\beta \gamma }{\theta }+\mu >\alpha ,\\ E_{3}=\left(I^{*},R^{*}\right)=\left(\sqrt{\frac{C}{\left(\beta \gamma /\theta \right)+\mu -\alpha }},\frac{\gamma }{\theta }\right). \end{array}$$

This equilibrium entails a situation whereby infected individuals, robots, and migration restrictions are present in the population. It is expected that infected individuals will be identified by the robots and the inflow of new infection will be stopped; hence, the disease spread can be drastically reduced.

**Theorem 3 .**

The equilibrium point *E*_2_ is locally asymptotically stable when *μ* > *α*.

Proof: The proof is trivial.

**Theorem 4 .**

The equilibrium point *E*_3_ is locally asymptotically stable when (*βγ*/*θ*) + *μ* > *α*.

**Proof**

Consider the Jacobian matrix of (4):

(17) $$\begin{array}{c} J\left(I,R\right)=\left[\begin{array}{cc} \alpha -\beta R-\frac{C}{I^{2}}-\mu & -\beta I\\ 0 & -\theta \end{array}\right] \end{array}$$

Substituting *E*_4_ in (17), we get

(18) $$\begin{array}{c} J\left(I^{*},R^{*}\right)=\left[\begin{array}{cc} \left(\alpha -\beta \frac{\gamma }{\theta }-\mu \right)-\frac{C}{{\left(\sqrt{C/\left(\left(\beta \gamma /\theta \right)+\mu -\alpha \right)}\right)}^{2}} & -\beta \sqrt{C/\left(\left(\beta \gamma /\theta \right)+\mu -\alpha \right)}\\ 0 & -\theta \end{array}\right],\\ J\left(I^{*},R^{*}\right)=\left[\begin{array}{cc} 2\left(\alpha -\beta \frac{\gamma }{\theta }-\mu \right) & -\beta \sqrt{C/\left(\left(\beta \gamma /\theta \right)+\mu -\alpha \right)}\\ 0 & -\theta \end{array}\right]. \end{array}$$

The characteristic equation of the Jacobian matrix is

(19) $$\begin{array}{c} \lambda^{2}+t_{r}\left(J\right)\lambda +\det\left(J\right)=0,\\ t_{r}\left(J\right)=2\left(\alpha -\beta \frac{\gamma }{\theta }-\mu \right)-\theta . \end{array}$$

Since we already assume (*βγ*/*θ*) + *μ* > *α*, then

(20) $$\begin{array}{c} \alpha -\frac{\beta \gamma }{\theta }-\mu <0. \end{array}$$

Therefore,

(21) $$\begin{array}{c} 2\left(\alpha -\beta \frac{\gamma }{\theta }-\mu \right)-\theta <0,\\ \det\left(J\right)=2\left(\alpha -\beta \frac{\gamma }{\theta }-\mu \right)\left(-\theta \right)-0\\ =-2\left(\alpha -\beta \frac{\gamma }{\theta }-\mu \right)\theta . \end{array}$$

Since 2(*α* − *β*(*γ*/*θ*) − *μ*) < 0 and −*θ* < 0, then

(22) $$\begin{array}{c} -2\left(\alpha -\beta \frac{\gamma }{\theta }-\mu \right)\theta >0. \end{array}$$

Note that the steady state solution in which the infected individuals are absent is not considered here, because it is not biologically important.

## 4. Numerical Simulations

**Figure 1:**
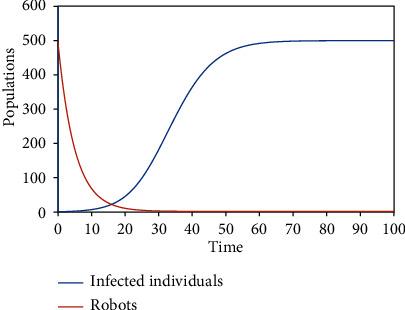
The dynamics of the infected individuals when there are robots for identification of the infected individuals but there is no restriction on migration.

**Figure 2:**
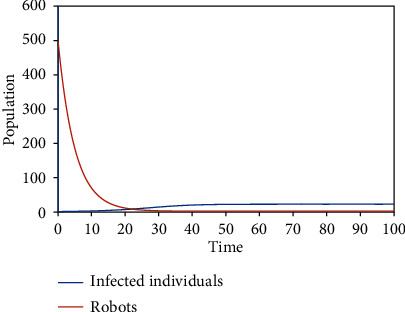
The dynamics of the infected individuals when there are robots for identification of the infected individuals and there is restriction on migration.

**Figure 3:**
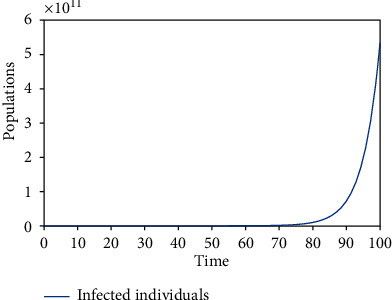
The dynamics of the infected individuals when there are no robots for identification of the infected individuals but there is restriction on migration.

**Figure 4:**
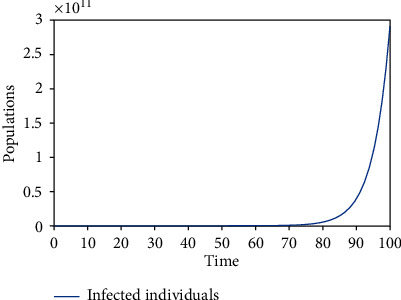
The dynamics of the infected individuals when there are no robots for identification of the infected individuals and there is no restriction on migration.

Numerical simulations using the variable and parameter values as given in Table 2 were carried out. Simulations for the various equilibrium solutions in Section 3 were given.  Figure 1 gives the dynamics of the infected individuals when there are robots for identification of the infected individuals but there is no restriction on migration, which is equivalent to *E*_1_.  Figure 2 gives the dynamics of the infected individuals when there are robots for identification of the infected individuals and there is restriction on migration, which is equivalent to *E*_3_.  Figure 3 gives the dynamics of the infected individuals when there are no robots for identification of the infected individuals but there is restriction on migration, which is equivalent to *E*_2_. Finally, Figure 4 gives the dynamics of the infected individuals when there are no robots for identification of the infected individuals and there is no restriction on migration, which is equivalent to *E*_0_.

From the graphs, we can see that the best policy is to adopt the robot identification and migration restriction policies together as illustrated in Figure 2. From Figure 1, even though there is robot identification policy but with time due to the increase in the number of migrants, the number of infected individuals shoots up.  Figure 3 shows that the restriction of migration policy alone cannot be used to curtail the spread of the disease. Figure 4 shows that the situation becomes worst when there are no robots for identification and there is no restriction on migration.

## 5. Summary and Conclusion

In this paper, we used a mathematical modeling approach to study the effectiveness of some of the strategies adopted by policy makers to curtail the spread of COVID-19. Two compartmental models were constructed. The first model considered migration into the infective population, while the second model considered migration restriction.

In the first model, two equilibrium solutions were obtained: *E*_0_ and *E*_1_. *E*_0_ is the solution obtained when there are infected individuals in the population but there are no robots to identify them. *E*_1_ is the solution obtained when there are infected individuals in the population and there are robots to identify them. Local stability analyses of the equilibria were proved. Figures 4 and 1 show the numerical results corresponding to  *E*_0_ and *E*_1_, respectively.

In the second model, two equilibrium solutions were also obtained: *E*_2_ and *E*_3_. *E*_2_ is the solution obtained when there are infected individuals and migration restrictions in the population. *E*_3_ is the solution obtained when there are infected individuals, migration restrictions, and robots in the population. Local stability analyses of the equilibria were proved. Figures 3 and 2 show the numerical results corresponding to *E*_2_ and *E*_3_, respectively.

From the graphs, one can conclude that the best policy is to adopt the robot identification and migration restriction policies together as illustrated in the second graph.

## Figures and Tables

**Table 1:** Meaning of variables and parameters.

Variable/parameter	Meaning
*R*	Robot population
*I*	Infected human population
*C*(*I*)	Migration factor into infected human population
*α*	Rate at which the population of infected individual increases
*γ*	Production rate of robots
*θ*	Rate at which robots stop functioning
*β*	Rate at which robots detect the infected individual
*μ*	Death rate of the infected individual due to COVID-19

**Table 2:** Variable and parameter values.

Variable/parameter	Value
*I*	600
*R*	500
*C*	100
*α*	0.5
*μ*	0.3
*γ*	0.4
*θ*	0.2

## Data Availability

There is no data available for this research.
